# Getting the best outcomes from epilepsy surgery

**DOI:** 10.1002/ana.25205

**Published:** 2018-04-10

**Authors:** Vejay N. Vakharia, John S. Duncan, Juri‐Alexander Witt, Christian E. Elger, Richard Staba, Jerome Engel

**Affiliations:** ^1^ Department of Clinical and Experimental Epilepsy UCL Institute of Neurology London United Kingdom and Chalfont Centre for Epilepsy; ^2^ Department of Epileptology University of Bonn Medical Center Bonn Germany; ^3^ David Geffen School of Medicine University of California Los Angeles, Los Angeles CA

## Abstract

Neurosurgery is an underutilized treatment that can potentially cure drug‐refractory epilepsy. Careful, multidisciplinary presurgical evaluation is vital for selecting patients and to ensure optimal outcomes. Advances in neuroimaging have improved diagnosis and guided surgical intervention. Invasive electroencephalography allows the evaluation of complex patients who would otherwise not be candidates for neurosurgery. We review the current state of the assessment and selection of patients and consider established and novel surgical procedures and associated outcome data. We aim to dispel myths that may inhibit physicians from referring and patients from considering neurosurgical intervention for drug‐refractory focal epilepsies. Ann Neurol 2018;83:676–690

Surgery is effective for many patients with drug‐resistant epilepsy (DRE), but it is underutilized.^1^ After a randomized trial of surgery for temporal lobe epilepsy (TLE) 2 reported 64% seizure freedom, surgery was recommended as the treatment of choice for drug‐resistant TLE.^3^ A subsequent trial of surgery for drug‐resistant TLE of <2 years reported 85% seizure freedom, and improved quality of life and socialization.^4^

In the USA, <1% of patients with continuing seizures are referred to epilepsy centers.^1^ The delay from onset of epilepsy to surgery averages >20 years, resulting in impaired social and educational development.^5^ Early surgery provides the best opportunity for seizure remission, minimizing adverse social and psychological consequences and premature death.

## Reasons for Underutilization of Epilepsy Surgery

Common reasons for rejecting surgery include fear of complications, expense, and reservations about benefits. In actuality, morbidity and mortality from recurrent seizures are much greater than from surgical treatment,^6^ and the cost is considerably less than that of a lifetime of disability.^7^ The delay to surgical referral has not changed in recent years,^8^, ^9^ and referrals to epilepsy surgery centers have decreased.^10^ This may be due to a decrease in mesial TLE, and because some patients are having surgery at low‐volume hospitals, where outcomes are less good. Of patients who undergo phase 1 noninvasive evaluation (magnetic resonance imaging [MRI], language functional MRI [fMRI], neuropsychology, neuropsychiatry, scalp video‐electroencephalography [EEG]) after an outpatient screening visit at an epilepsy surgery center, it may be expected that half will not proceed further, 25 to 40% may be recommended for resection without further investigation, and 10 to 25% will require intracranial EEG.

A likely reason for underreferral is misconceptions held by nonspecialist physicians (Table 1). It has been suggested, therefore, that surgery per se should not be emphasized as a reason for referral, but all patients with refractory epilepsy merit review at an epilepsy center, where there are a range of treatment options, including consideration of surgery.^1^

**Table 1:** Common Misconceptions about Epilepsy Surgery

Misconception	Fact
All drugs need to be tried.	The chance of seizure remission is <10% after 2 drugs have failed.
Bilateral EEG spikes are a contraindication to surgery.	Patients with unilateral seizure onset commonly have bilateral interictal spikes.
Normal MRI is a contraindication to surgery.	Other techniques often detect a single epileptogenic zone in patients with normal MRIs.
Multiple or diffuse lesions on MRI are a contraindication to surgery.	The epileptogenic zone may involve one or part of a lesion.
Surgery is not possible if eloquent cortex is involved.	The risk–benefit ratio can be individually evaluated.
Surgery will make memory worse if there is an existing memory deficit.	Poor memory usually will not get worse and may improve.
Chronic psychosis is a contraindication to surgery.	Patients may benefit if seizures are eliminated.
IQ < 70 is a contraindication to surgery.	Individuals with IQ < 70 may benefit from remission or reduction in seizures.

EEG = electroencephalography; IQ = intelligence quotient; MRI = magnetic resonance imaging.

## Patient Selection

Mesial TLE is the prototypical surgically remediable epilepsy syndrome. Others include discrete neocortical lesions such as focal cortical dysplasias and diffuse lesions limited to one hemisphere. Excellent outcomes can also be achieved in patients with multiple lesions, for example, tuberous sclerosis when one tuber is the source of seizures. The best prognostic factor for a good outcome is a discrete structural lesion on MRI, in an area that can be safely removed, which conforms to the location of ictal EEG changes and is consistent with seizure semiology. Conversely, the occurrence of generalized tonic–clonic seizures, a normal MRI, extratemporal onset, psychiatric comorbidity, and learning disability reduce the chances.^11^

## Measuring Outcome

The success of surgery for DRE is assessed by postoperative seizures and effects on health‐related quality of life (HRQOL). There are 2 seizure outcome scales. In the Engel scale, the seizure‐free category can be divided into continued auras and aura‐free.^12^ The International League against Epilepsy (ILAE) scale considers seizure freedom, continued auras, and postoperative seizures in terms of improvement or lack thereof from the preoperative seizure status with a 6‐point scale.^13^ These scales are not so useful for patients with severe epilepsies when the intervention is palliative and it can be ascertained what the patients or their caregivers expect to gain from surgery. A “contract” is then made and success or failure determined by whether the contract is fulfilled.^14^ Seizure outcome needs to be considered in the long term, with the recognition that there is an attrition rate of seizure freedom over the following 10 to 20 years, and some may remit after some years.^11^

Evaluation of epilepsy surgery usefully includes HRQOL measures that determine effects on school and work performance, domiciliary arrangements, driving, interpersonal relationships, memory, and other cognitive functions.

## Presurgical Investigations

### Evolving Role of Brain Imaging

Presurgical evaluation aims to localize the epileptogenic zone (EZ) that must be removed to give seizure freedom through the integration of seizure semiology, EEG, neuropsychological evaluation, and multimodal imaging. Patients with an identified epileptogenic lesion have 2.5 times higher odds of seizure freedom following surgery than those without.^15^ Advances in MRI, nuclear medicine, and source localization techniques can help to improve delineation of the EZ. Surgically remediable lesions include developmental abnormalities, infections, neoplasia, stroke, trauma, and vascular malformations.^16^ Optimized imaging acquisition and interpretation increase the detection of epileptogenic lesions (Table 2).^17^

**Table 2:** Imaging Sequences Commonly Employed for Presurgical Evaluation

MRI	3D volumetric T1‐weighted imaging (1mm isotropic voxels) in AC‐PC angulation
	T2‐weighted axial and coronal images (<3mm slice thickness) angulated perpendicular to hippocampal axis
	3D volumetric FLAIR (1mm isotropic voxels) or axial and coronal images (<3mm slice thickness) angulated perpendicular to hippocampal axis
	T2* gradient echo or susceptibility‐weighted axial imaging angulated perpendicular to hippocampal axis
Confirmation of epileptogenic zone	^18^F‐FDG PET
	Ictal interictal subtraction SPECT
	MEG
	Electrical source imaging
	EEG‐fMRI
Eloquent function mapping	Language and motor functional MRI
	Tractography
	Transcranial magnetic stimulation

F‐FDG = ^18^F‐fluorodeoxyglucose; 3D = 3‐dimensional; AC‐PC = anterior and posterior commissure; EEG = electroencephalography; FLAIR = fluid‐attenuated inversion recovery; fMRI = functional MRI; MEG = magnetoencephalography; MRI = magnetic resonance imaging; PET = positron emission tomography; SPECT = single photon emission computer tomography.

### MRI

**Figure 1:**
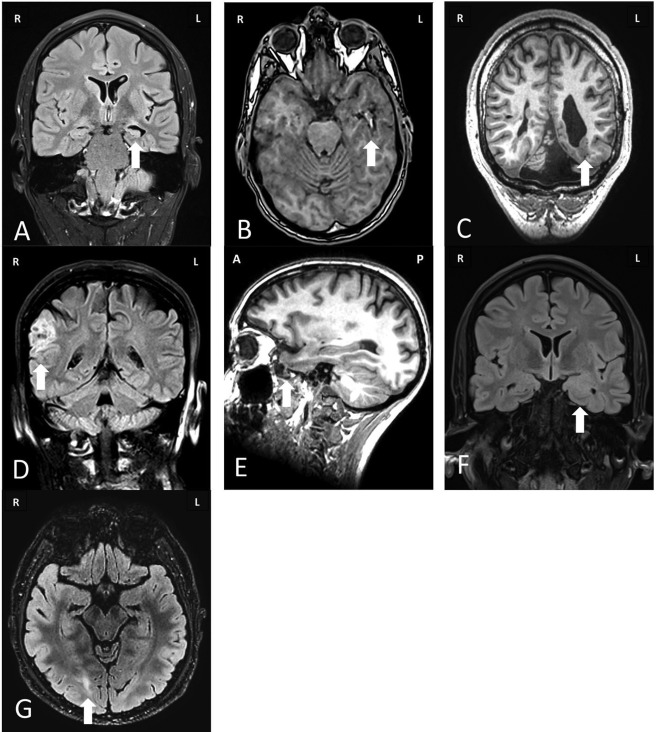
Magnetic resonance imaging of common pathologies underlying drug‐resistant focal epilepsy that are amenable to surgical treatment. (A) Coronal fluid‐attenuated inversion recovery (FLAIR) image showing increased T2 signal in the left hippocampus associated with volume loss and compensatory dilatation of the left temporal horn consistent with left hippocampal sclerosis. (B) Nonenhanced axial T1‐weighted image of a patient with a lesion in the left temporal lobe that has a “popcorn” appearance due to a hemosiderin ring and mixed intensity blood products consistent with a cavernoma. (C) Nonenhanced coronal T1‐weighted image of a patient with multiple bilateral well‐demarcated periventricular lesions that have imaging characteristics matching gray matter consistent with nodular periventricular heterotopia. This is associated with polymicrogyrialike overlying cortex. Note is also made of a posterior fossa arachnoid cyst and ventricular asymmetry. (D) Coronal FLAIR image of a patient with a sharply demarcated cortically based “pseudocystic” lesion in the right supramarginal gyrus that returns a hyperintense signal, consistent with a dysembryoplastic neuroepithelial tumor. There is associated overlying calvarial remodeling. (E) Sagittal T1‐weighted image through the left temporal lobe revealing herniation of the temporal pole through the floor of the middle cranial fossa consistent with a meningoencephalocele. (F) Coronal FLAIR image with increased signal and expansion of the left amygdala. Contrast‐enhanced imaging did not reveal any enhancement, consistent with a diffusely infiltrating low‐grade glioma. (G) Axial FLAIR image revealing increased signal in the right occipital lobe with blurring of the cortical–subcortical margin consistent with type 2B focal cortical dysplasia.

Epilepsy imaging protocols have been suggested by the ILAE,^18^ and optimal MRI protocols have been defined.^19^ The mainstay is high‐quality structural 3T MRI (Fig 1). This provides higher identification of lesions than 1.5T scans.^20^

Volumetric T1‐weighted gradient‐recalled echo (GRE) images provide sharp gray/white matter distinction for detection of subtle malformations of cortical development. One‐millimeter isotropic voxels allow reformatting in additional planes and segmentation of the hippocampus for volumetric measurements that may identify subtle atrophy and determine the structural integrity of the contralateral hippocampus.^21^ Gadolinium enhancement is recommended when tumors, infection, or neurocutaneous syndromes are suspected.^22^

High‐resolution T2‐weighted coronal images acquired in a plane perpendicular to the long axis of the hippocampus give optimal resolution. T2* sequences such as GRE or susceptibility‐weighted images improve the detection of calcified or hemorrhagic lesions.

Hippocampal sclerosis is the most commonly identified pathology in surgical series and is characterized by hippocampal atrophy and increased T2 signal intensity. Visual inspection can miss subtle, focal, or bilateral hippocampal sclerosis. T2 relaxometry quantifies the T2 relaxation time along the length of the hippocampus. Quantification increases detection of hippocampal sclerosis.^23^ Three‐dimensional (3D) T2‐weighted fluid‐attenuated inversion recovery sequences may detect focal cortical dysplasias at the bottom of a sulcus, with blurring of the gray–white boundary and dyslamination extending into the white matter. Postacquisition processing of MRI may increase detection of subtle abnormalities, but reduced specificity is the price of increased sensitivity.^24^, ^25^, ^26^

If a lesion is detected that is concordant with clinical semiology, with interictal and ictal video‐EEG, and with satisfactory neuropsychological and neuropsychiatric assessments, no further investigation may be required before definitive surgery. If the planned resection margins are close to eloquent cortex, functional mapping such as language and motor fMRI and transcranial magnetic stimulation may help to delineate resection boundaries.^27^

The commonly used fMRI language paradigms verbal fluency and verb generation lateralize, rather than precisely localize, language functions. The sensitivity and specificity of fMRI for language lateralization is between 80 and 90% and has replaced the intracarotid sodium amobarbital procedure in most cases.^27^

Tractography is derived from diffusion‐weighted MRI sequences and can localize major white matter tracts such as the corticospinal tract and optic radiation. Tractographic and dissection studies have shown considerable variability in the anterior extent of Meyer's loop, ranging from 20 to 50mm from the temporal pole.^28^ Anterior temporal lobe resections may cause visual field deficits that preclude up to 50% of patients from driving even if they are seizure‐free.^29^ Presenting the tractographic representation of the optic radiation into the surgical microscope eyepiece during temporal lobe resection prevented visual field defects.^30^

Novel MRI contrasts may identify covert lesions. Diffusion kurtosis imaging provides improved gray–white matter contrast and may act as a biomarker for severity and disease subtypes.^31^

### Nuclear Medicine

If MRI does not identify a lesion that is concordant with clinical and EEG data, functional imaging with positron emission tomography (PET) or single photon emission computer tomography (SPECT) may be useful.

PET imaging is generally performed interictally, due to the short unpredictable nature of spontaneous seizures, identifying hypometabolism as a marker of cortical dysfunction. PET MRI provides better anatomical and functional information than PET computed tomography.^32^ Interictal PET has a sensitivity of up to 90% in temporal and 50% in extratemporal lobe epilepsy.^33^ The region of hypometabolism detected by ^18^F‐fluorodeoxyglucose (^18^F‐FDG) PET is generally larger than the EZ and cannot be used to outline a surgical resection plan. ^18^F‐FDG PET may aid hemispheric lateralization and general lobar localization in cases with discordant scalp EEG and/or normal MRI. The overall positive predictive value of a good outcome following ^18^F‐FDG PET in TLE was 77.5% when MRI, EEG, or both were nonconcordant. Specific PET ligands for γ‐aminobutyric acid type A, N‐methyl‐D‐aspartate, opioid, and serotonin receptors have research applications but are not in widespread use.^34^

SPECT imaging utilizes technetium‐99m–labeled ligands to measure regional cerebral blood flow (rCBF). The tracer can be administered at the time of seizure onset and will be distributed in the brain to reflect rCBF at the time of injection. Tracer administration as early after seizure onset as possible is crucial to identify hyperperfusion associated with the seizure onset zone (SOZ). Delayed administration visualizes areas that show hyperperfusion due to seizure propagation. Ictal SPECT had a 70% sensitivity compared to 78% with interictal ^18^F‐FDG PET.^35^ When ictal and interictal SPECT are normalized and subtracted, the sensitivity of SPECT has reached 87%.^36^

### Other Functional Imaging Methods

Simultaneous scalp EEG‐fMRI can show hemodynamic changes associated with interictal epileptic discharges with 30 to 40% sensitivity^37^ and may assist planning intracranial implantations,^38^ with widespread abnormalities associated with poor outcome from resection.^39^ Ictal EEG‐fMRI often shows widespread hemodynamic changes before the onset of the seizure on scalp EEG,^40^ highlighting the low sensitivity of scalp EEG. The current clinical place of scalp EEG‐fMRI is visualization of ictal and interictal networks that may help design intracranial EEG strategies and indicating whether there is likely to be a poor outcome, which may justify stopping further investigation.

Magnetoencephalography (MEG) measures the magnetic field generated by synchronized postsynaptic currents in cortical pyramidal cell dendrites. In magnetic source imaging, current dipole maps of interictal spikes are overlaid onto an MRI scan. The spatial and temporal resolution of MEG is superior to scalp EEG but is limited to dipoles on the cortical surface and less sensitive to deeper sources. In patients in whom the MEG signal was concordant with the resection, long‐term seizure outcome was Engel I in 85%, compared to 37% when the MEG signal was not concordant.^41^ Thus, MEG has a limited role in helping to define the EZ.

In clinical practice, EEG‐fMRI, MEG, and electrical source imaging (ESI) map interictal epileptic discharges, with a small chance of recording seizures; this chance is greater with ESI, because prolonged recordings are possible. Patients who may benefit from these investigations are those who require intracranial EEG to define the EZ. These data may help generate a hypothesis to test with intracranial EEG and to identify patients with widespread abnormalities, who should not proceed.

### 3D Visualization

**Figure 2:**
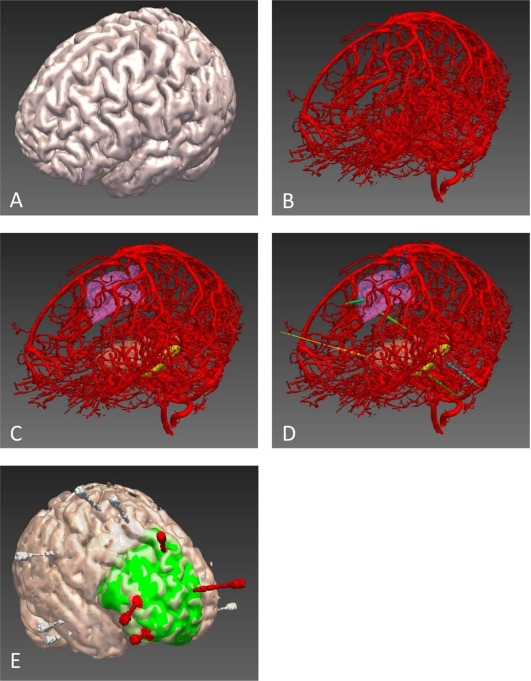
(A) Three‐dimensional cortical model. (B) Vascular segmentation from digital subtraction angiogram following left internal carotid and vertebral injections. (C) Vascular segmentation with automated parcellation of anatomical regions of interest (ROIs), including supplementary motor cortex, anterior insula, and hippocampus. (D) Automated electrode trajectory placement targeting predefined anatomical ROIs. Not all of the implemented electrode trajectories or target ROIs are shown in this image. (E) Postimplantation reconstruction of a different patient to that shown in A–D. Bolt and electrode contact points are segmented from postimplantation computed tomography and overlaid on cortical model. Electrodes with contacts implicated at seizure onset are shown in red, whereas those not involved at seizure onset are shown in white. Planned resection volume to include electrodes at seizure onset is shown in green. For clarity, the bolts are displayed, not the individual electrode contact points.

The 3D visualization of multimodal imaging demonstrates the spatial relationships of normal and abnormal structures and function in the brain. This is beneficial for planning intracranial EEG placements and resection. Computer‐assisted planning of electrode insertion and resection planning promise to simplify the epilepsy surgery pathway (Fig 2).^42^, ^43^

### Place and Process of Intracranial EEG

Intracranial EEG recordings are performed to: (1) localize the site of ictal onset when the hypotheses for location of the EZ are reasonably limited, (2) define the extent of the EZ when a tailored resection is required, and (3) map essential cortical functions adjacent to contemplated areas of resection.

Intracranial recordings include intraoperative electrocorticography, and chronic extraoperative recording using subdural or intraparenchymal depth electrodes.

In 1993, most centers agreed that intraoperative electrocorticography, or chronic subdural grid or strip recordings, were appropriate for a suspected EZ on the cortical convexity, whereas depth electrodes were more appropriate for a suspected EZ in deeper structures.^44^ Subdural grids or stereotactically placed EEG (SEEG) recordings were necessary for functional mapping and determination of the extent of the EZ to guide tailored resection. The consensus was that both methods are useful for neocortical EZ and both have limitations, the choice being individualized for each patient.

There are no definitive criteria for determining the EZ boundaries. The location of interictal spikes may help but is often unreliable. The location of the ictal onset is important but does not determine the extent of the EZ. There is a need for biomarkers that delineate the EZ. Pathological high‐frequency oscillations (pHFOs) may serve this purpose.

There is a strong association between occurrence of pHFOs and epileptogenic tissue.^45^ Although invasive EEG is currently the gold standard for recording pHFOs, new MEG methods could help detection.^46^

Poor seizure outcome was associated with high “nonharmonicity” on the postresection electrocorticogram, which implies that residual tissue can generate epileptiform activity.^47^ Good seizure outcome has been predicted by a combination of low interictal EEG synchrony outside the seizure onset zone (SOZ) and low delta power (0–4Hz) inside the SOZ.^48^ Good seizure outcome was predicted by removal of highly epileptogenic areas derived from mathematical models of interictal EEG functional connectivity.^49^

### Accuracy and Method of SEEG

The overall morbidity rate from SEEG has been reported as 1.3% per patient, equating to a risk of 1 of 287 electrodes. Hemorrhage occurred in 1% of patients.^50^ Methods used to detect intracranial vasculature are varied. Some units use contrast enhanced magnetic resonance (MR) venography and angiography, whereas others perform digital subtraction catheter angiography (DSCA). DSCA is the gold standard but is invasive and may require a general anaesthetic.^51^ Proponents of MR venography do not consider the additional vasculature visualized with DSCA to be clinically relevant and have not reported increased hemorrhage rates.^52^

Planning SEEG electrode placement is time‐consuming and requires a multidisciplinary approach to ensure adequate sampling of regions consistent with the electrophysiological hypothesis. Generally, SEEG electrodes are planned to enter the brain on the crown of a gyrus, maximize distance from cerebral vasculature, not transgress sulcal pial boundaries, not come within 10mm of other implanted electrodes, cross the skull orthogonally, have the shortest feasible intracranial length, and maximize gray matter contact.

Cardinale et al suggested a 3mm safety margin from cerebral vasculature.^53^ Computer‐assisted planning algorithms increase safety and reduce the time taken (see Fig 2).^42^, ^54^, ^55^ Implantation methods for SEEG include frame‐based, frameless, and robotic systems. Bone‐anchored fiducials are more accurate than scalp fiducials or surface tracing registration methods. Frameless techniques are quicker than frame‐based systems, especially when 8 to 14 SEEG electrodes are inserted, at the relative cost of accuracy. Robotic systems allow highly accurate electrode placement with shorter implantation times than frame‐based or frameless systems. Accuracy data have been published for Neuromate, ROSA, and iSYS1. A meta‐analysis of accuracies of implantation methods revealed significant heterogeneity between studies, mainly due to use of different accuracy measures.^56^ Robotic guidance achieved a median 0.78mm entry point and 1.77mm target point error, compared to a median 1.43mm entry point and 2.69mm target point error with manual Talairach frame placement.^53^

## Scope of Surgical Treatment

### Types of Surgical Procedures

Some resections may be standardized when the EZ is within recognized boundaries, for example anterior temporal resections for mesial TLE, and hemispherectomies for diffuse lesions. Neocortical resections are usually tailored based on electrophysiological and imaging data constrained by proximity to eloquent cortex, such as language or motor areas. Palliative procedures include disconnection, such as corpus callosotomy, for disabling drop attacks. Palliative neuromodulation includes vagus nerve stimulation, deep brain stimulation,^57^ and responsive neurostimulation and are considered when there is not a single, removable EZ.^58^ These may reduce seizure frequency and severity, but very rarely bring seizure freedom.^59^

#### Temporal Lobe Resection

The most common anterior temporal resection procedure includes resection of up to 4.5cm neocortex, measured from the temporal pole to minimize visual and speech deficits, and an en bloc resection of the amygdala, hippocampus, parahippocampus, uncus, and fusiform gyrus via the temporal horn.^60^ In a meta‐analysis, anterior temporal lobectomy had an 8% greater seizure freedom rate than transcortical selective amygdalohippocampectomy.^61^ Amygdalohippocampectomy was associated with better postoperative memory than temporal pole and hippocampal resection.^62^

#### Frontal Lobe Resection

Frontal lobe resections account for up to 30% of cases and carry a 1‐year seizure remission rate of approximately 45% (range = 21–61%) and less durable long‐term outcomes.^63^ The EZ frequently extends beyond MRI‐defined lesions, and the resection may need to be tailored according to invasive EEG findings.^64^ The best postoperative outcome is associated with type 2B focal cortical dysplasia, a focal seizure onset, and total resection of the EZ.^63^, ^64^

#### Insula Resection

Seizure remission rates of 60 to 70%^65^ and 84% in a series of insular tumors^66^ have been reported. Insula resections, without a well‐defined lesion on MRI, require a careful analysis of the risk–benefit ratio, especially in the language‐dominant hemisphere.

#### Parietal Lobe Resection

Parietal seizures may have few localizing semiological features but can present with somatosensory disturbances, vertigo, psychic symptoms, and language dysfunction.^67^ Propagation to the frontal lobes results in hyperkinetic seizures, and spread to the temporal lobe causes automatisms. Engel I outcomes range between 45 and 78%, with the best being associated with a focal MRI lesion.^68^

#### Occipital Lobe Resection

Scalp EEG demonstrated occipital interictal spikes in only 17%.^69^ Resection had an average Engel I outcome in 65% (range = 20–100%).^70^ A discrete MRI lesion and age < 18 years were predictive of successful surgical outcome. Occipital lobe epilepsy surgery carries significant risk of postoperative visual dysfunction.

#### Functional Hemispherectomy

When the EZ is extensive in one hemisphere, hemispherotomy, or functional hemispherectomy, may be considered. Generally, this is restricted to individuals who have a hemiparesis with loss of meaningful hand function.^71^ Seizure freedom occurred in 73%.^72^ Most patients who are walking prior to surgery remain so afterward. There is loss of any fine motor skills in the contralateral upper and lower limbs, whereas cognitive outcomes are usually stable, with language functions having developed in the contralateral hemisphere.^72^

#### Corpus Callosotomy

Corpus callosotomy is a palliative procedure for patients with generalized epilepsy or diffuse bilateral or unilateral origin with rapid propagation. Corpus callosotomy can be either anterior, posterior, or total.^73^ Anterior callosotomy may be performed first and converted to a total callosotomy if disabling seizures continue. A posterior callosotomy spares interfrontal connections.^74^ Meta‐analyses have shown a 59% seizure reduction after anterior compared to 88% after total corpus collosotomy.^73^

### Novel Techniques

#### Stereotactic Radiosurgery

Seizure‐free outcomes range from 0 to 86%, with a mean Engel I outcome of 51%.^75^ Headache and cerebral edema are common, frequently there is a transient increase in focal seizures in the months following treatment, and there is the possibility of radionecrosis.^76^ The ROSE (Radiosurgery or Open Surgery for Epilepsy) trial compared gamma knife and surgery for mesial temporal lobe epilepsy.^77^ The trial stopped early because of poor recruitment, and results are awaited. Further uses of stereotactic radiosurgery include corpus callosotomy and treatment of hypothalamic hamartomas.^78^

#### Laser‐Induced Thermal Therapy

**Figure 3:**
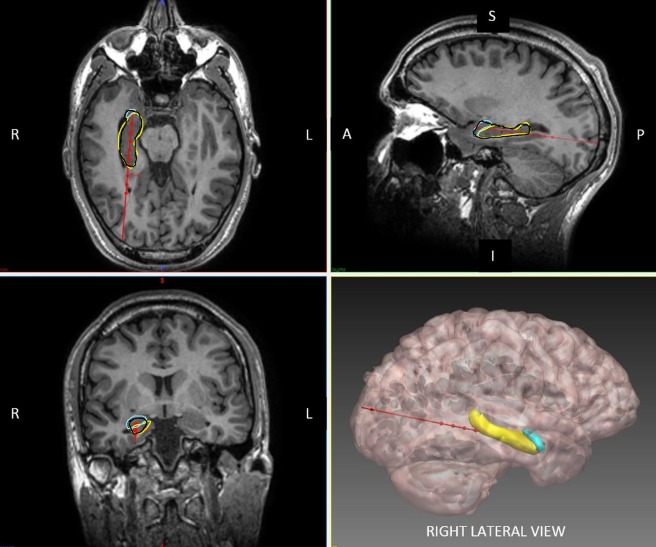
Axial, sagittal, coronal, and 3‐dimensional reconstruction of planned laser ablation trajectory (red) with outline of hippocampus (yellow), amygdala (cyan), and modeled ablation cavity (black). Other structures, such as the entorhinal cortex and parahippocampal gyrus, have been excluded for clarity. The entry point of the trajectory is centered over the crown of a gyrus, parallel to the superficial sulci. The ideal trajectory should maximize distance from vasculature, avoid crossing sulci or the lateral ventricle. In this example, the entry point is within the inferior occipital gyrus and the target point is on the anterior border of the amygdala. The Visualase (Medtronic) system is capable of performing an ablation diameter of between 5 and 20mm. The modeled ablation cavity shown above is based on a conservative estimate of 15mm.

MR‐guided laser‐induced thermal therapy (MgLiTT) can produce a 5‐ to 20mm‐diameter focal ablation zone. Heating is monitored using real‐time MR thermography, allowing precise control of the ablation zone. Two systems are in use, Visualase (Medtronic, Plymouth, Minnesota) and NeuroBlate (Monteris Medical, Minneapolis, MN). The Visualase system has been used for hypothalamic hamartoma (HH), focal cortical dysplasia,^79^, ^80^ periventricular heterotopia,^81^ and mesial temporal lobe epilepsy (MTLE).^82^ Sixty‐five percent of MTLE had an Engel I outcome at 1‐year follow‐up.^83^ As with SEEG electrodes, laser trajectories should avoid cerebral vasculature, pial sulcal boundaries, and the lateral ventricle, and maximize ablation of the hippocampus and amygdala. Computer‐generated trajectories improve ablation volume and calculated trajectory risk compared to when manually planned (Fig 3).^84^, ^85^ In a nonrandomized comparison, MgLiTT gave similar outcomes to conventional surgery.^86^ MgLiTT may result in less adverse cognitive effects, is repeatable, does not preclude subsequent surgery, and may represent a minimally invasive first‐line treatment for MTLE. Laser ablation of HH resulted in 86% seizure freedom at a mean 9‐month follow‐up and no permanent complications.^87^

#### MR‐Guided Ultrasound

MR‐guided focused ultrasound (MRgFUS) ablation is a minimally invasive method of creating focal lesions.^88^ The lesion extent can be monitored using MR thermography. The development of 1,000‐array element transducers, active scalp cooling, and improved focusing has produced a resurgence in interest in transcranial MRgFUS for treating brain tumors and epilepsy and performing functional procedures.

### Neurological and Surgical Complications of Epilepsy Surgery

Neurological complications of epilepsy surgery depend on the extent and location of the surgical resection and preexisting functional deficits and are governed by hemispheric language dominance, vascular injury, and proximity to critical white matter tracts and eloquent cortex.

In a Cochrane review, the reported overall complication rate was 7.3%.^89^ Few studies report pre‐ and postoperative neuropsychological assessment of cognition, language, memory, social function, visual fields, and psychiatric sequelae. Reported complication rates were higher in patients older than 50 years and range from 6 to 25%.^90^ A review of almost 1,000 patients at a single institution found the overall complication rate was 17%,^91^ with unanticipated long‐term new neurologic deficits in 3%. The most common complication was a permanent visual field deficit, sufficient to preclude driving, in 9.4% following temporal lobe resection. The incidence of infection requiring bone flap removal was 2.6%. The risk of infection was 4‐fold higher in those having subdural EEG grid placement prior to resection.

## Neuropsychological Evaluation and Epilepsy Surgery

### Cognitive Deficits in Epilepsy

Neuropsychological impairments in epilepsy are the result of the underlying pathology, seizures, interictal epileptic discharges, antiseizure drugs, and psychiatric comorbidities.^92^ The impact of these factors depends on their onset in relation to brain maturation, cognitive development, and the brain's ability to compensate for adverse impacts. Compensatory mechanisms can maintain cognitive functions that would have otherwise been compromised.

### Neuropsychological Tests—Why, How, Sensitivity, and Confounders

Neuropsychological evaluation is an essential component of presurgical evaluation, providing valuable information on lateralized and localized brain dysfunctions.^93^

Compensatory mechanisms can result in “discordant” findings. For example, an early onset lesional epilepsy may cause transfer of verbal functions to the originally nondominant hemisphere, thereby suppressing nonverbal functions.^92^

fMRI and the intracarotid amobarbital procedure^92^ can lateralize language and memory functions. The latter is now reserved for rare circumstances, when structural and fMRI and neuropsychological data do not give clarity on the capacity of the contralateral hemisphere. Corticographic mapping of language functions becomes relevant when eloquent cortex is at risk.

The preoperative neuropsychological profile is important when advising the patient about the cognitive risks of the proposed surgery, and for estimating the functional integrity of the structures to be resected and the reserve capacities of the remaining brain.^92^

Neuropsychological assessment depends on the use of measures that are sensitive to detect lateralized and localized brain dysfunctions associated with epilepsy and its underlying pathology. Given the high prevalence of TLE, the validity of test measures has mostly been demonstrated for left (verbal learning and memory, naming) and less consistently for right (nonverbal/visual–spatial learning and memory) temporal lobe functions.^92^ Prefrontal deficits (attention, executive functions, working memory, and motor coordination) can be assessed, although lateralization is challenging.^92^ An evidence‐based assessment of parietal and occipital epilepsies has not been established, because experience with these epilepsies is low.

Tests must be suitable for longitudinal reassessments (sufficient retest reliability, parallel versions, and test–retest norms). Surgical reference centers are currently attempting to standardize assessments and provide evidence‐based recommendations for test selection.^94^

It is essential to consider the adverse cognitive effects of medication^93^ and recent seizures. Furthermore, psychiatric comorbidities may adversely affect test performance,^95^ and these should be assessed by screening measures.^96^

### Determinants of Postoperative Outcome

Preserving cognition is a high priority. Inevitably, surgical procedures carry a risk of cognitive deterioration. However, seizure control and reducing medication can lead to cognitive improvement. The determinants of neuropsychological outcome after epilepsy surgery are summarized in Table 3.

**Table 3:** Determinants of Neuropsychological Outcome after Surgical Treatment of Epilepsy

Type and quality of surgery
Extent, side, and site of surgery
Degree of actual selectivity in terms of sparing functional tissues beyond the epileptogenic lesion
Collateral damage
Complications
Functional integrity of resected (and surgically affected) tissues
As estimated by the degree of pathology and the respective presurgical neuropsychological performance
Individual reserve capacities
Functional integrity of the remnant brain or homologue contralateral structures
Degree of functional plasticity
Age at surgery
Gender
Postsurgical control of epileptic activity
Epileptic seizures
Interictal epileptic discharges
Changes in antiepileptic treatment
Quantitative and qualitative changes of the drug regimen
Implantation of intracranial depth electrodes within subsequently nonresected tissues

Collateral damage and complications can have detrimental effects on cognition.^97^ Thus, more selective procedures (such as stereotactic thermocoagulation) are valued from a neuropsychological perspective.

An important determinant of neuropsychological outcome is the functional integrity of the resected structures.^98^ Resecting dysfunctional tissue is associated with a low risk, whereas resecting functional tissues carries a high risk of cognitive deterioration. The functional integrity can be estimated by the neuropsychological deficits associated with the resection site, combined with imaging markers of its structural integrity.^99^ fMRI for episodic memory is emerging as a predictor of verbal memory decline after anterior temporal lobe resection,^100^ and the risk of word‐finding difficulty after dominant temporal lobe resection can be estimated with fMRI, but with less specificity.^101^ In children, verbal memory improved after right temporal lobe resection and visual memory improved after left temporal lobe resection, suggesting functional release of the contralateral side.^102^ After left temporal lobe resection, verbal memory is better preserved with smaller medial temporal resections,^102^ and the posterior hippocampus has a key role in preserving memory.^103^

Further determinants of neuropsychological outcome include the reserve capacity of the remnant brain, the degree of age‐dependent functional plasticity, postsurgical seizures, interictal epileptic discharges, and changes of antiseizure medication.^104^ Additionally, depth electrodes in subsequently nonresected tissues can negatively affect cognitive outcome.^105^

Factors increasing risk of an unfavorable cognitive outcome are: (1) an unimpaired neuropsychological profile; (2) no MRI lesion; (3) a very low presurgical performance, indicating a limited reserve; and (4) bilateral pathology such as bilateral hippocampal sclerosis.^106^ These must be considered when selecting and advising potential surgical patients about weighing the cognitive risks against the chance of seizure freedom.

## Psychiatric Antecedents and Sequelae of Epilepsy Surgery

Three major questions arise concerning psychiatric disturbances within the context of epilepsy surgery: (1) Does a past or current psychiatric condition affect the chances of seizure freedom? (2) Does epilepsy surgery increase risk of de novo psychiatric disturbances or an exacerbation of preexistent problems? and (3) Can epilepsy surgery resolve psychiatric problems?

Interictal depression (5–50%), anxiety (0–48%), interictal psychosis (0–16%), and suicidality are pressing issues in pharmacoresistant epilepsy patients.^107^ Contributory causes include structural brain damage, active epilepsy, and adverse side effects of antiseizure medication.^108^ Furthermore, psychiatric comorbidity may be reactive to the epilepsy and associated psychosocial difficulties.

Past or current psychopathology is associated with a lower chance of seizure freedom.^109^ This, however, is not a contraindication for surgery,^110^ and successful surgery may improve psychiatric symptoms, with improved depression and anxiety after TLE surgery. A short‐term increase in psychiatric symptoms (especially anxiety) may be followed by long‐term improvement.^107^

The most significant risk factor for postsurgical psychiatric problems is a presurgical affective disorder or a lifetime psychiatric diagnosis.^107^

De novo psychopathology has been observed in 1 to 26% of patients after TLE surgery (depression, 4–18%; anxiety, 3–26%; interictal psychosis, 1–12%^107^). De novo psychiatric problems are associated with a preoperative history of secondary generalized tonic–clonic seizures.^107^ The incidence of new psychogenic nonepileptic seizures following epilepsy surgery is estimated at 4%, being higher (8.5%) in females with a psychiatric history.^111^, ^112^

A systematic presurgical evaluation of psychopathology and postoperative follow‐up are appropriate, with consideration of the causes of the psychopathology, including organic, iatrogenic, and psychosocial aspects. Therapeutic options can include antiseizure drugs with positive psychotropic effects such as lamotrigine and avoiding drugs with negative psychiatric risks.^113^ In addition, antidepressants or neuroleptics, and psychotherapy can be used prior to and after surgery. Improvement of psychiatric symptoms can be a positive by‐product of epilepsy surgery, and there are some striking cases of resolution of antisocial behavior.^114^

## Social and Employment Consequences of Epilepsy Surgery

Although the primary reason for patients undergoing epilepsy surgery is seizure freedom, other aspects are also taken into account, including anticipated psychosocial and psychological improvements and a better quality of life.

Major concerns for epilepsy patients include the inability to drive, lack of independence, unemployment, embarrassment, stigma, medication side effects, and injuries.^115^ Patients expect improvements in these areas after successful treatment.

An adverse outcome after surgery, 8% in one study,^116^ would be unchanged or worsened seizure control accompanied by a decline in cognition. These “double losers” report the most severe deteriorations in HRQOL. In these patients, presurgical characteristics indicate a lower chance of postsurgical seizure freedom or a higher risk of cognitive decline,^116^ emphasizing the need for careful patient selection, comprehensive presurgical diagnostics, and advising patients about the chances and risks of surgery.

### Vocational Outcome

Data on vocational outcome after epilepsy surgery are sparse, and studies report inconsistent findings, with increased, unchanged, or decreased employment opportunities after surgery.^117^ Postsurgical seizure freedom, a younger age at surgery, and employment prior to surgery increase the likelihood of a favorable vocational outcome. A postsurgical rehabilitation program can improve employment status.^118^ Furthermore, the prevalent job market and social/welfare systems are potential confounders.

### Adjustment Issues—The Burden of Normality

Postsurgical seizure freedom can lead to adjustment issues, the so‐called “burden of normality.”^119^ Seizure relief may decrease dependency and the level of consideration displayed by others and increase expectations toward the now “cured” patient. This may pose considerable pressure on the seizure‐free patient, who is now abruptly forced to cope with this new situation. Such a constellation can overtax the patient, and cause depression and anxiety, and may destabilize a relationship that was predicated on one party being dependent on the other.

### Expectation Management

The various outcome scenarios should be discussed with patients prior to surgery. Psychosocial counseling is important for evaluating individual risks/benefits as well as the neuropsychological risks. Unrealistic expectations must be managed and the inevitable uncertainty of individual outcomes addressed. The prolonged nature of presurgical evaluation enables counseling the patient and their family on the possible sequelae of epilepsy surgery and how to manage these. Epilepsy surgery strives for seizure control, but it cannot guarantee a better life, even if seizure freedom is achieved.

## Evolving Practice of Epilepsy Surgery

As epilepsy surgery becomes more prevalent, it is frustrating that many patients wait years for referral to consider this option. There have been changes in case mix in large epilepsy surgery centers, with a reduction of the numbers of patients with hippocampal sclerosis and an increase in patients with complex epilepsies who require intracranial EEG, and who do not have optimal characteristics. It is crucial that epilepsy surgery centers are experienced and multidisciplinary, and having made large investments in the area, they must guard against the risk of overselling epilepsy surgery and must recognize the limitation of benefits and the potential adverse effects, and that some individuals are not going to be improved by having part of their brain removed.

Future challenges are first to ensure that all potential candidates for epilepsy surgery are evaluated in appropriate centers after failing to gain seizure control with 2 or 3 medications over 2 to 3 years. Second, noninvasive diagnostic procedures must be improved, so that those who will benefit from surgery are quickly selected and others are directed to other treatment options. Third, less invasive treatments must be devised, so that craniotomies become rarities. Fourth, rehabilitation must be improved, so that individuals and their families may capitalize on the benefits accrued by surgery.

## Author Contributions

J.S.D. conceived the article and its structure. V.N.V. and J.S.D. contributed the sections “Presurgical Investigations” and “Scope of Surgical Treatment.” J.‐A.W. and C.E.E. contributed the sections “Neuropsychological Evaluation and Epilepsy Surgery,” “Psychiatric Antecedents and Sequelae of Epilepsy Surgery,” and “Social and Employment Consequences of Epilepsy Surgery.” R.S. and J.E. contributed the sections “Reasons for Underutilization of Epilepsy Surgery” and “Place and Process of Intracranial EEG”.

## Potential Conflicts of Interest

Nothing to report.
